# Human-to-swine introductions and onward transmission of 2009 H1N1 pandemic influenza viruses in Brazil

**DOI:** 10.3389/fmicb.2023.1243567

**Published:** 2023-08-08

**Authors:** Dennis Maletich Junqueira, Caroline Tochetto, Tavis K. Anderson, Danielle Gava, Vanessa Haach, Maurício E. Cantão, Amy L. Vincent Baker, Rejane Schaefer

**Affiliations:** ^1^Laboratório de Bioinformática e Evolução de Vírus, Departamento de Bioquímica e Biologia Molecular, Centro de Ciências Naturais e Exatas (CCNE), Universidade Federal de Santa Maria (UFSM), Santa Maria, Brazil; ^2^Embrapa Suínos e Aves, Concórdia, Brazil; ^3^Virus and Prion Research Unit, United States Department of Agriculture, National Animal Disease Center, Agricultural Research Service, Ames, IA, United States; ^4^Laboratório de Virologia, Departamento de Microbiologia, Imunologia e Parasitologia, Instituto de Ciências Básicas da Saúde, Universidade Federal do Rio Grande do Sul (UFRGS), Porto Alegre, Brazil

**Keywords:** influenza A virus, 2009 H1N1, swine, spillover, Brazil

## Abstract

**Introduction:**

Once established in the human population, the 2009 H1N1 pandemic virus (H1N1pdm09) was repeatedly introduced into swine populations globally with subsequent onward transmission among pigs.

**Methods:**

To identify and characterize human-to-swine H1N1pdm09 introductions in Brazil, we conducted a large-scale phylogenetic analysis of 4,141 H1pdm09 hemagglutinin (HA) and 3,227 N1pdm09 neuraminidase (NA) gene sequences isolated globally from humans and swine between 2009 and 2022.

**Results:**

Phylodynamic analysis revealed that during the period between 2009 and 2011, there was a rapid transmission of the H1N1pdm09 virus from humans to swine in Brazil. Multiple introductions of the virus were observed, but most of them resulted in self-limited infections in swine, with limited onward transmission. Only a few sustained transmission clusters were identified during this period. After 2012, there was a reduction in the number of human-to-swine H1N1pdm09 transmissions in Brazil.

**Discussion:**

The virus underwent continuous antigenic drift, and a balance was established between swine-to-swine transmission and extinction, with minimal sustained onward transmission from humans to swine. These results emphasize the dynamic interplay between human-to-swine transmission, antigenic drift, and the establishment of swine-to-swine transmission in shaping the evolution and persistence of H1N1pdm09 in swine populations.

## Introduction

In 2009, an influenza A virus (IAV) containing a unique combination of genes not previously identified in animals or humans emerged, causing the first pandemic of the 20th century with widespread global morbidity and mortality (Viboud et al., 2010). It was later determined that the 2009 pandemic H1N1 virus (H1N1pdm09) was the result of reassortment events among North American triple-reassortant swine IAV lineage (segments PB2, PB1, PA, HA, NP, and NS) and Eurasian avian-like swine H1N1 lineage (M and NA) (Garten et al., 2009). Though the H1N1pdm09 virus was not detected in pigs until after its emergence in humans (Chen et al., 2013; Watson et al., 2015; Rajão et al., 2017), reassorted viruses that share a common ancestor with the H1N1pdm09 were detected in central Mexico several years before its identification in humans (Smith et al., 2009; Mena et al., 2016).

Upon emergence in the human population, H1N1pdm09 was rapidly introduced back into swine on several different occasions around the world (Nelson et al., 2012, 2015c; Vincent et al., 2013; Ciacci-Zanella et al., 2015; Pippig et al., 2016; Chastagner et al., 2018). Human-to-swine IAV introductions and subsequent sustained onward transmission between pigs have been shown to be more frequent than swine-to-human transmission (Nelson et al., 2012; Nelson and Vincent, 2015; Rajão et al., 2017). Since 2009, our understanding of the global diversity and evolution of IAV in swine increased to address paucity in data and concerns about the risk of interaction among H1N1pdm09 and enzootic IAV in pigs, and ultimately on how these viruses impact human health (Ninomiya et al., 2002; Smith et al., 2009; Anderson et al., 2013; Vincent et al., 2013; Nelson et al., 2014). The expansion of IAV surveillance allowed a better understanding of the genetic diversity of IAV in swine, revealing the impact of frequent introductions of human viruses on IAV evolution in swine (Pereda et al., 2010; Vijaykrishna et al., 2010; Ducatez et al., 2011; Chen et al., 2013; Nelson et al., 2014, 2015a,c,d; Pippig et al., 2016; Fraiha et al., 2021).

Swine herds in Brazil have recognized outbreaks of acute respiratory disease associated with H1N1pdm09 infections since 2010 (Schaefer et al., 2011; Rajão et al., 2013; Schmidt et al., 2016; Ferreira et al., 2022). These outbreaks were located primarily in the South, Midwest, and Southeast regions associated with intensive swine production (Nelson et al., 2015b). Subsequently, surveillance efforts for detection of IAV in swine populations have increased in the last decade, especially through the efforts made by the Brazilian Agricultural Research Corporation (EMBRAPA Swine and Poultry). As a result, at least eight introductions of human seasonal H1N1, H1N2, and H3N2 viruses were detected in swine in Brazil, along with H1N1pdm09 (Tochetto et al., 2023). Most of these introductions had sustained onward transmission in swine prior to 2009, followed by reassortment with H1N1pdm09 lineage viruses. These events contributed to significant increases in genomic diversity (Nelson et al., 2015b; Fraiha et al., 2021; Tochetto et al., 2023).

Serological and molecular studies demonstrated that H1N1pdm09 viruses are endemic with variable prevalence from year to year in swine in Brazil (Ciacci-Zanella et al., 2015; Dias et al., 2015; Fraiha et al., 2021). However, little information has been gathered about the population dynamics of the H1N1pdm09 lineage in Brazil. To understand the evolution of H1N1pdm09 in the Brazilian swine herds and to fully characterize human-to-swine introductions, we conducted a large-scale phylogenetic analysis of swine H1N1pdm09 viruses collected between 2009 and 2020.

## Materials and methods

### Sample preparation and sequencing

Between 2009 and 2020, nasal swabs and lung samples collected from swine showing respiratory clinical signs in the Brazilian states of Minas Gerais, Paraná, Rio Grande do Sul e Santa Catarina were sent to a private diagnostic laboratory for viral screening. IAV-positive samples were then sent to the virology laboratory at EMBRAPA Swine and Poultry for virus isolation in SPF embryonated chicken eggs and/or MDCK cells (Zhang and Gauger, 2014) and sequencing. Viral isolation was confirmed by RT-qPCR after two viral passages. Total viral RNA was extracted, and the HA and NA gene segments were amplified by RT-PCR using SuperScript^™^ III One-Step RT-PCR System with Platinum^™^ Taq DNA Polymerase (Invitrogen^™^; Thermo Fisher Scientific^®^, Waltham, MA, United States) following manufacture’s guideline (PCR amplification of influenza A genomic segments for whole-genome sequencing, Ion Torrent sequencing application guide; Thermo Fisher Scientific^®^, Waltham, MA, United States). DNA libraries were prepared and submitted for sequencing using Ion Torrent system (Thermo Fisher Scientific^®^, Waltham, MA, United States). Influenza segments were assembled using Newbler v.2.9 (Roche, United States). In total, 70 H1 hemagglutinin and 55 N1 neuraminidase unique gene sequences were generated for this study.

### Sequence data

The HA and NA sequences generated in this study were combined with the 100 most similar sequences identified using BLASTn (Altschul et al., 1990) and GISAID (Shu and McCauley, 2017). Additionally, human and swine South American IAVs, reference sequences associated with the H1pdm09 (Mena et al., 2016) and the World Health Organization (WHO)-recommended human seasonal H1 vaccine strains were downloaded from the Influenza Research Database (Zhang et al., 2017), NCBI GenBank or the GISAID EpiFlu database and included in the final dataset. Each segment was aligned separately in MAFFT v7.490 (Katoh and Standley, 2013). The Swine H1 Classification Tool from the BV-BRC and preliminary phylogenetic trees were used to recognize and exclude non-pandemic strains among HA and NA datasets. We excluded identical sequences, except for those obtained from swine sampled in different regions. Final alignments included 4,141 H1pdm09 sequences (1,701 base pairs, bp) and 3,227 N1pdm09 sequences (1,410 bp) isolated from humans or swine between 2009 and 2022 (Supplementary Material S1).

### Phylogenetic analysis and reassortment detections

Maximum likelihood (ML) trees incorporating the general time-reversible (GTR) model of nucleotide substitution (Tavaré, 1986) with a gamma-distributed rate variation (Γ) and invariant sites (Yang, 1993) were inferred for HA and NA using IQ-TREE v1.6.12 (Vienna, Austria) (Nguyen et al., 2015). The best-fitting model of nucleotide substitution for each alignment was determined prior to phylogenetic reconstruction (Kalyaanamoorthy et al., 2017). The statistical support for branches within the inferred trees was assessed using SH-like approximate Likelihood-Ratio Test (SH-aLRT) (Guindon et al., 2010) and Ultra-Fast Bootstrapping (UFB) approximation with 1,000 pseudoreplicates (Hoang et al., 2018). For visualization, the phylogenetic trees were rooted on A/California/07/2009(H1N1) and plotted in FigTree v1.4.4 (Rambaut, 2009). Human-to-swine H1N1pdm09 transmission events were explicitly defined using the inferred phylogenetic tree topology. To delineate clades representing sustained transmission in swine following spillover, specific criteria were applied as follows: (a) monophyletic clades exclusively composed of swine sequences isolated in Brazil, (b) clades exhibiting branch support SH-aLRT/UFB > 90, and (c) clades comprising ≥3 sequences obtained in at least two distinct years.

To explore potential reassortment events involving H1N1pdm09 in swine populations of Brazil, we performed ML tree reconstructions for H1pdm09 and N1pdm09. Specifically, we included sequences obtained exclusively from this study, employing the same parameters as described above. Reassortment events were identified by assessing phylogenetic incongruence in gene trees using dendextend v1.17.1 package (Galili, 2015) in R v4.2.1 (RStudio Team, 2019; R Core Team, 2020).

### Temporal dynamics

To estimate the time of the most recent common ancestor (tMRCA) of each human-to-swine transmission clade in Brazil, reduced datasets were assembled using TARDiS (Marini et al., 2022). This algorithm kept swine strains isolated in Brazil and then optimized genetic diversity and temporal signal while subsampling the HA and NA datasets to a computationally feasible size of 100 and 89 sequences, respectively. Each of these alignments was screened for incongruent divergence and sampling dates in TempEst v1.5.3 and to quantify temporal signal (Rambaut et al., 2016). Swine sequences from each human-to-swine transmission clade identified in the ML tree were combined with the subsampled datasets obtained in TARDiS. The HA and NA were analyzed in BEAST v1.10.4 (Suchard et al., 2018) implementing an uncorrelated relaxed molecular clock and a non-parametric Bayesian Skyline demographic model (Drummond et al., 2005) with 10 piecewise-constant skyline groups. Based on preliminary results, a normal distribution with mean 4 × 10^−3^ and 3 × 10^−3^ mutations/site/year for H1pdm09 and N1pdm09, respectively, was used in configuring the priors for the mean evolutionary rate. MCMC chains were run for 3.0 × 10^8^ chain steps, with subsampling every 30,000 iterations. The BEAGLE library was used to improve computational performance (Ayres et al., 2012). Convergence of runs was evaluated in TRACER v1.7.2 (Rambaut and Drummond, 2007) based on the Effective Sample Sizes of each estimated parameter (ESS > 200) after excluding an initial 10% of the iterations as burn-in. Statistical support was reflected by values of the 95% highest posterior density (95% HPD). A summary Maximum Clade Credibility tree (MCC) was inferred in Tree Annotator v1.10.4 and viewed using Figtree v1.4.4 (Rambaut, 2009).

To provide estimates of relative diversity and the effective population size of H1N1pdm09 IAV in swine, all swine H1pdm09 and N1pdm09 sequences from Brazil were independently combined and analyzed in BEAST v1.10.4. The coalescent-based Gaussian Markov random field (GMRF) Bayesian Skyride method (Minin et al., 2008) with the time-aware smoothing parameter under an uncorrelated relaxed lognormal molecular clock and the GTR + Γ + I substitution model were used. MCMC chains were run for 4.0 × 10^8^ chain steps, with subsampling every 40,000 iterations. Parameter convergence was assessed using TRACER v1.7.2 (Rambaut and Drummond, 2007) and the GMRF Skyride reconstruction plot was visualized using R v4.2.1 (RStudio Team, 2019; R Core Team, 2020).

### Natural selection

To test for signals of selection acting on H1N1pdm09 IAVs circulating in swine in Brazil, representative datasets for both humans (500 random sequences from Brazil) and swine (all available sequences from Brazil) were assembled for HA and NA genes. Additionally, swine sequences were divided into two datasets according to the isolation year (2009–2011 and 2012–2020) to assess the adaptive evolution process of H1N1pdm09 IAVs in Brazil across the time. Global non-synonymous to synonymous (dN/dS) rate ratios were estimated for each dataset with the SLAC method (Kosakovsky Pond and Frost, 2005) in HyPhy v2.2.4 (Philadelphia, United States) (Pond et al., 2005). The MEME method (Murrell et al., 2012) in DataMonkey (Weaver et al., 2018) was then used to identify the positively selected sites for each dataset with the significance level at *p* < 0.05. A matrix of pairwise genetic distances from HA and NA gene sequences was generated and plotted for each dataset using the ape package in R v4.2.1 (RStudio Team, 2019; R Core Team, 2020).

## Results

### Human-to-swine H1N1pdm09 transmission events

**Figure 1:**
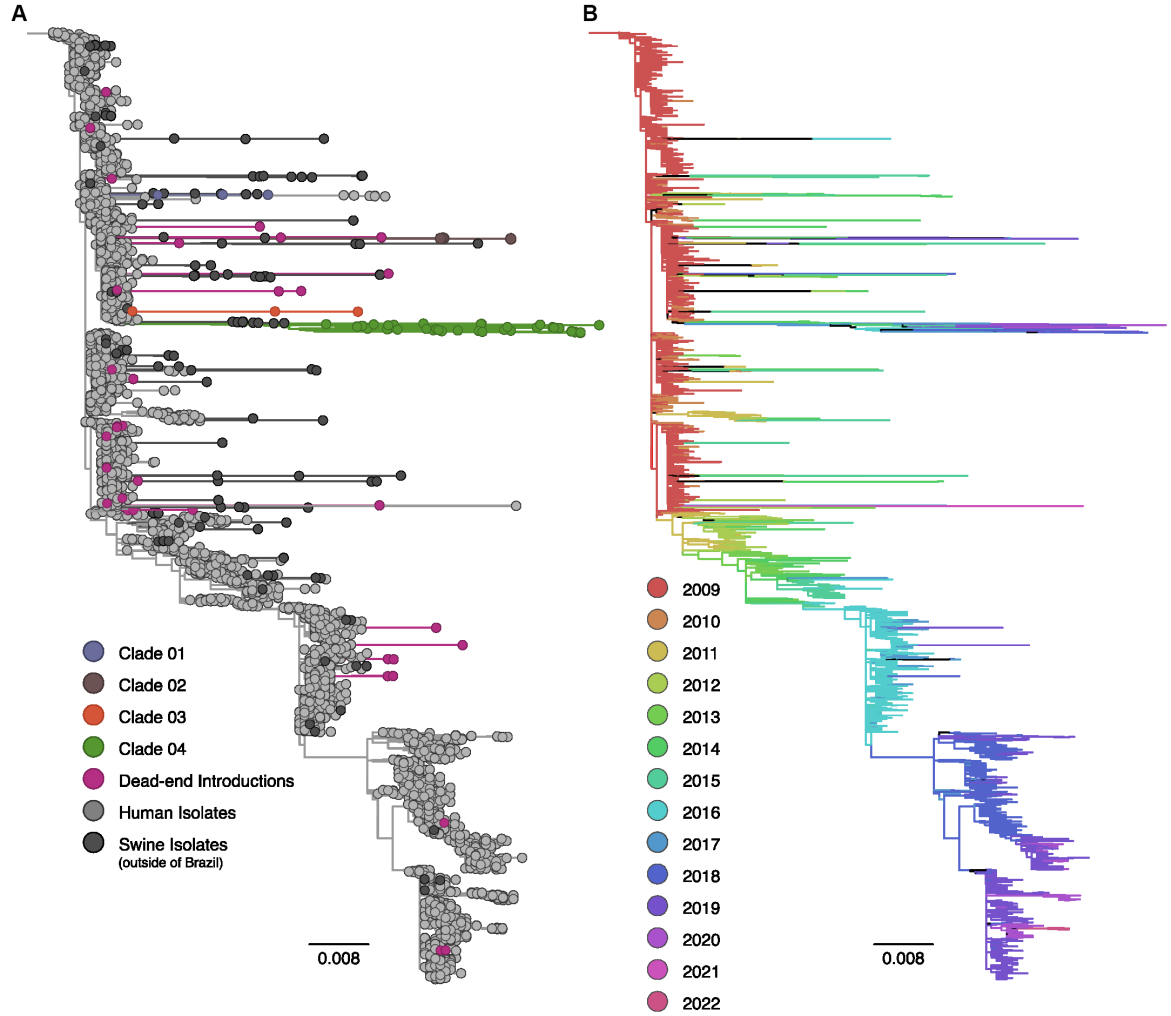
Maximum likelihood phylogenetic tree inferred for 4,141 human and swine influenza A H1pdm09 hemagglutinin gene sequences collected worldwide from 2009 to 2022. **(A)** Phylogenetic tree indicating introductions of human H1pdm09 into swine in Brazil. The clades labeled 01 to 04 encompass monophyletic clades including three or more swine isolates (sampled in at least two different years) and suggest sustained H1N1pdm09 transmission following spillover to swine. Dead-end introductions are colored violet and have no evidence for onward transmission in swine. Sequences isolated from swine in Brazil are colored according to the figure key, human strains and swine strains isolated globally are also indicated. **(B)** The same phylogeny depicted in **(A)** with branches colored by year of sampling. Branches lengths are drawn to scale with the scale bar reflecting nucleotide substitutions per site; the tree is rooted on A/California/07/2009(H1N1).

**Figure 2:**
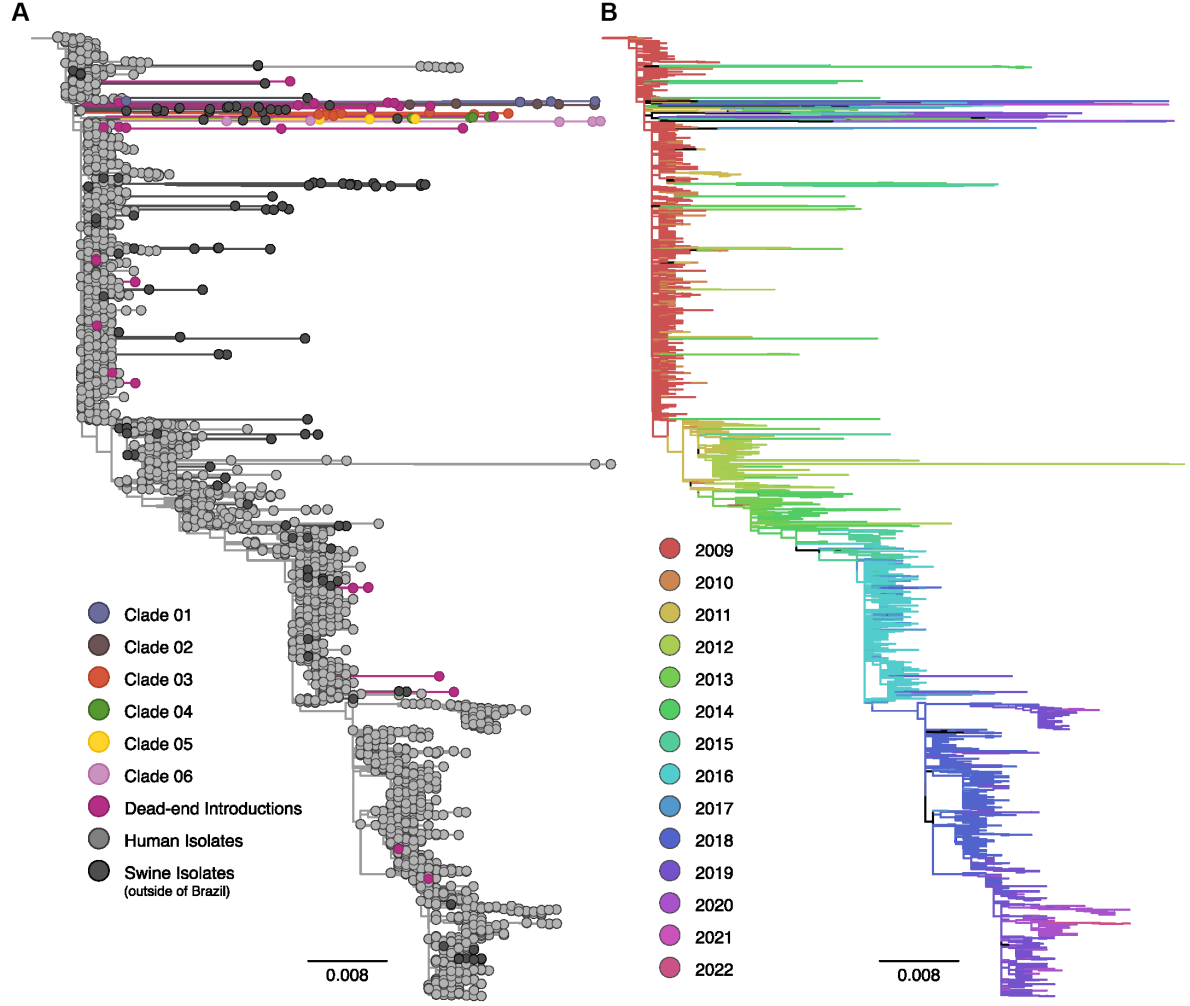
Maximum likelihood phylogenetic tree inferred for 3,227 human and swine influenza A N1pdm09 neuraminidase gene sequences collected worldwide from 2009 to 2022. **(A)** Phylogenetic tree indicating introductions of human H1pdm09 into swine in Brazil. The clades labeled 01 to 06 encompass monophyletic clades including three or more swine isolates (sampled in at least two different years) and suggest sustained H1N1pdm09 transmission following spillover to swine. Dead-end introductions are colored violet and have no evidence for onward transmission in swine. Sequences isolated from swine in Brazil are colored according to the figure key, human strains and swine strains isolated globally are also indicated. **(B)** The same phylogeny depicted in **(A)** with branches colored by year of sampling. Branches lengths are drawn to scale with the scale bar reflecting nucleotide substitutions per site; the tree is rooted on A/California/07/2009(H1N1).

The phylogenetic analyses of HA and NA segments of the H1N1pdm09 lineage exhibited a temporal structure based on sampling dates (Figures 1, 2). Sequences isolated in 2009 displayed short internal branches near the root, indicating a rapid increase in genetic diversity with rapid transmission during the early phase of the pandemic. However, starting from 2012, the H1N1pdm09 virus likely underwent continuous antigenic drift, resulting in a characteristic ladder-like topology (Ma et al., 2020).

The phylogenetic analysis provided robust evidence of recurrent human-to-swine transmission events in Brazil, supporting the occurrence of at least 30 distinct introductions for each viral segment (Supplementary Table S1). Most of these human-to-swine transmissions are related to human samples collected in 2009–2011, implying a rapid spillover of the virus from humans to swine. Based on the predominance of singleton swine sequences in addition to the small clusters of two or three isolates, our analysis suggests that these introductions are likely indicative of self-limited infections in swine, with minimal or no subsequent onward transmission (dead-end introductions). Conversely, in select cases, the H1N1pdm09 continued to be successfully transmitted onward in swine as substantiated by the clustering of monophyletic clades encompassing ≥3 swine sequences isolated in Brazil over multiple years. All those clusters of transmission are phylogenetically related to strains circulating in humans in 2009. In nearly all cases, the swine HA and NA sequences collected in Brazil exhibited long branch lengths, indicating high genetic divergence and limited sampling of H1N1pdm09 viruses in both pigs and humans in Brazil.

In the context of the HA segment, 86.7% (*N* = 26) of the 30 human-to-swine spillover events resulted in dead-end transmissions. However, four introductions were suggestive of sustained transmission following spillover. Within these four clusters, the majority of the sequences were found within a single clade (Clade 04, *N* = 42); which included all the most recent H1pdm09 data collected in Brazil (*N* = 4, isolation year: 2020). In addition to the inclusion of 41 swine sequences, Clade 04 encompasses an isolate from a human case in Brazil (H1N2v), providing evidence of a previously documented swine-to-human transmission event in 2020 (World Health Organization (WHO), 2020). Following Clade 04, Clade 02 comprised five sequences isolated between 2017 and 2019, while both Clade 01 and Clade 03 consisted of three sequences.

For the NA phylogenetic tree, we identified six events of introduction with subsequent onward transmission in pigs (20%), as well as 24 events of dead-end introductions (80%, Figure 2). Clade 03 represented the largest cluster, including 13 sequences, followed by Clade 04 (*N* = 6, 2017–2019), Clade 01 (*N* = 5, 2010–2019), Clade 02 (*N* = 5, 2019–2021), and Clade 06 (*N* = 5, 2012–2020). Clade 05 was the smallest cluster, comprising only three sequences isolated between 2012 and 2015. Clade 02 also contained a human variant sequence (H1N1v) isolated in Brazil in 2021, indicating a separate swine-to-human transmission event.

**Figure 3:**
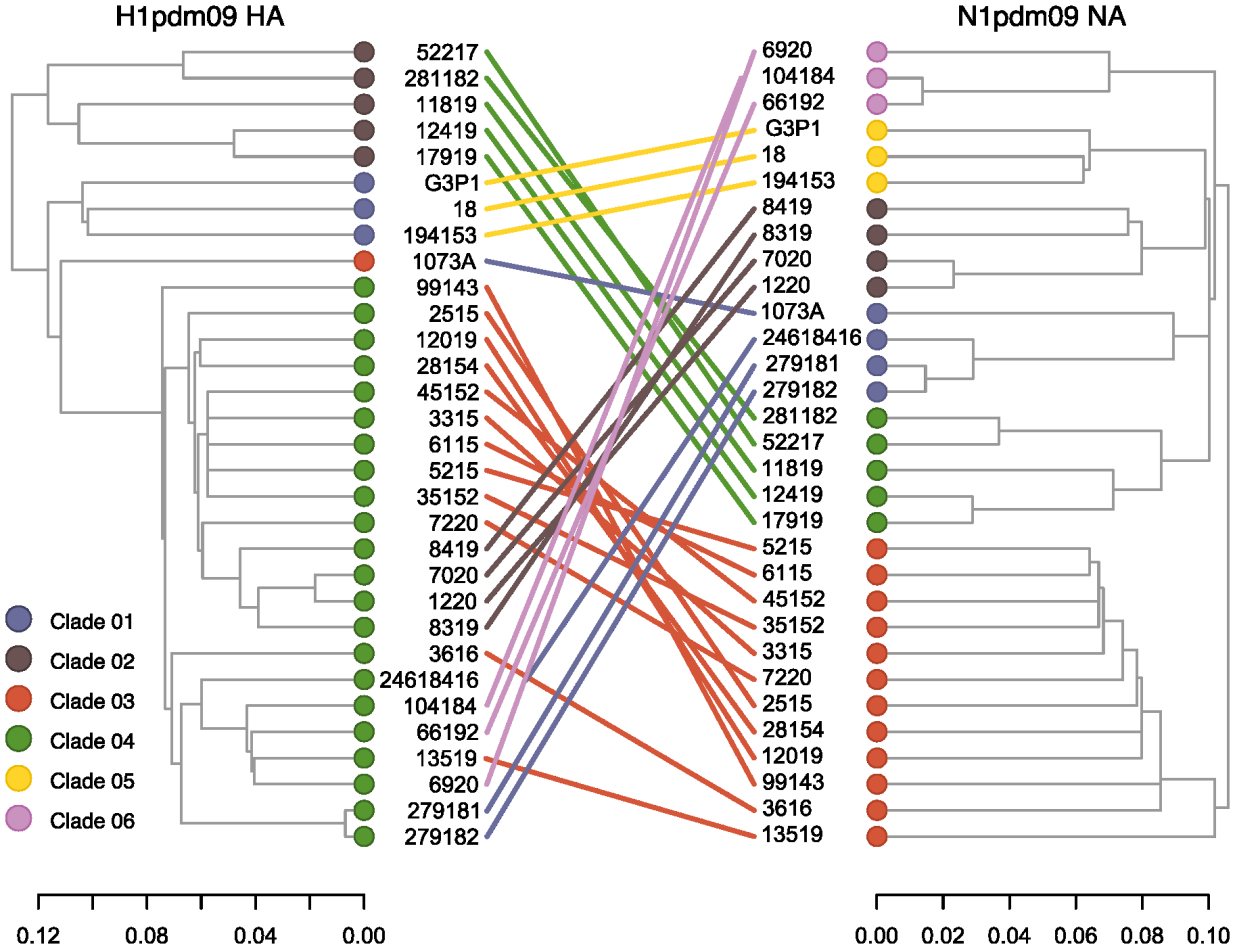
Tanglegram of swine H1pdm09 and N1pdm09 sequences isolated in Brazil. Corresponding taxa in the two trees are connected by a line. The connecting lines are colored by the N1pdm09 corresponding clade. Only samples with HA and NA segments fully sequenced were included in this analysis.

Reassortment events between HA and NA strains circulating among swine in Brazil were assessed through phylogenetic inference (Figure 3). We found consistent topologies between HA Clade 01 and NA Clade 05, as well as between HA Clade 02 and NA Clade 04. However, notable incongruences were observed for sequences within the HA Clade 04, which exhibited pairings with sequences from different NA clades (Clade 01, Clade 02, Clade 03, and Clade 06). Notably, one NA sequence within Clade 01 exhibited a pairing with HA Clade 03, rather than Clade 04. These results indicate the occurrence of reassortment events among H1N1pdm09 viruses circulating in swine in Brazil. Furthermore, while HA demonstrated lower diversification, greater diversification was observed within the NA segment.

### H1N1pdm09 population dynamics in swine in Brazil

**Figure 4:**
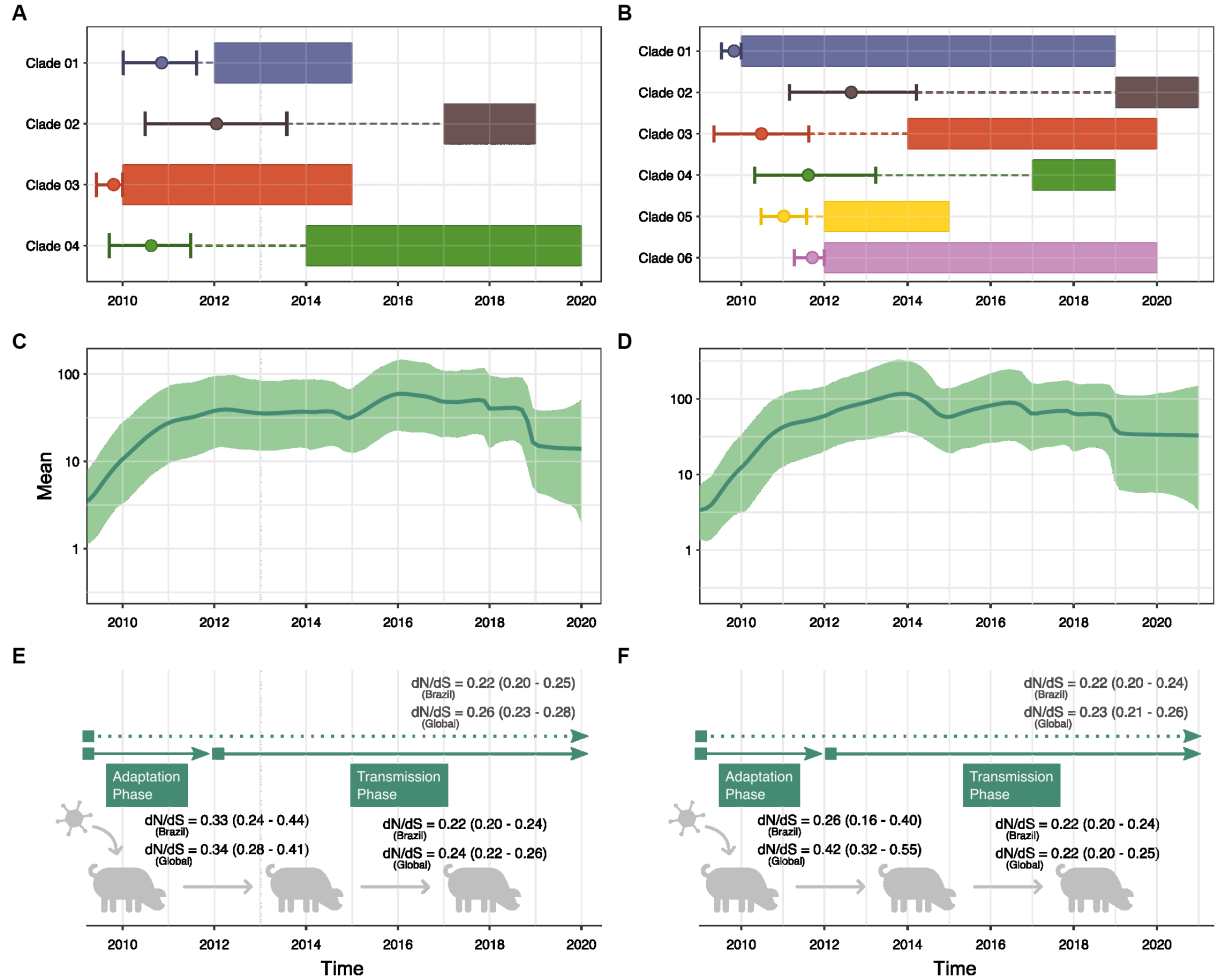
Demographic history reconstruction of human-to-swine H1N1pdm09 transmissions in Brazil. Bayesian reconstruction of the time to the most recent common ancestor of each clade of swine sequences isolated in Brazil within the H1pdm09 HA **(A)** and N1pdm09 NA **(B)** phylogenetic trees. The rectangles represent the time interval in which the samples were collected. The circle marks the Tmrca reconstruction, and the vertical lines show the confidence interval. Dashed lines show the time interval between Tmrca and the beginning of detection of the clade in Brazil. Bayesian Skyride analysis of H1pdm09 HA **(C)** and N1pdm09 NA **(D)** swine sequences isolated in Brazil. Thick solid line indicates the mean estimates for the effective population size (Ne) and green-shaded area indicate the 95% HPD interval. Estimates of global dN/dS ratios (*p* < 0.05) for H1pdm09 **(E)** and N1pdm09 **(F)** swine strains isolated in Brazil and in other countries (global) in two periods: 2009–2011 and 2012–2020.

Given the frequency of interspecies transmission, we applied a more thorough phylodynamic analysis to gain deeper insights into the evolution of H1N1pdm09 in swine. MCC phylogenies were inferred for all HA (*N* = 4) and NA (*N* = 6) clades identified in the ML trees. The obtained phylogenies exhibited a general agreement with the ML topology, revealing that the majority of persistent introductions into swine originated from common ancestors transmitted from humans between 2009 and 2011 (Figures 4A,B). Bayesian reconstructions suggest that HA Clade 01, Clade 03 and Clade 04 likely emerged in swine before 2012 and were detected until 2015, 2019 and 2020, respectively (based on sampling dates). Similarly, NA Clade 01, Clade 03, Clade 04, Clade 05, and Clade 06 also emerged before 2012 and persisted in swine for several years. Notably, sequences belonging to Clade 01 seem to have circulated in swine for approximately 9 years. Only one HA clade (Clade 02) and one NA clade (Clade 02) evolved from a common ancestor introduced in 2012, and no introductions resulting in onward transmission were observed beyond this period.

Through GMRF skyride coalescent model we inferred the past population growth dynamics of H1N1pdm09 circulating among pigs in Brazil (Figures 4C,D). Analyses of H1pdm09 and N1pdm09 demonstrated an exponential increase in the effective population size (Ne) until 2012, coinciding with the period of predominant human-to-swine transmissions (exponential phase). Subsequently, relative genetic diversity stabilized, suggesting a balance between successful swine-to-swine transmissions and extinctions (stable phase). Additionally, a minor decrease in relative genetic diversity as observed from 2019 onwards.

### Selective pressure on H1N1pdm09 circulating in swine

To assess H1N1pdm09 selective pressure in Brazil, we analyzed human and swine alignments separately. Selection analyses revealed that HA and NA strains isolated from swine in Brazil (2009–2020) had lower global dN/dS rate ratios than strains isolated from humans (2009–2021), implying stronger selection pressure acting on human viruses isolated in Brazil (Table 1), especially for NA. However, these results should be interpreted with caution for HA given that confidence intervals are overlapping. On the contrary, sequences isolated from swine in Brazil presented higher median pairwise genetic distance than the human dataset (Supplementary Figure S1), although the number of sequences analyzed may affect these results.

**Table 1:** Comparison of selection pressures according to the date of collection for H1pdm09 and N1pdm09.

Gene	Dataset	Dates	*N*	Global dN/dS (SLAC)	Positive selection (SLAC)	Positive selection (MEME)
				Mean (95% CI)	*p* < 0.05	*p* < 0.05
HA	Human (Brazil)*	2009–2021	500	0.243 (0.227, 0.261)	101, 158, 202, 203, 239	49, 158, 177, 203, 220, 239, 510, 551
	Swine (Brazil)	2009–2020	91	0.225 (0.205, 0.246)	159	87, 114, 159, 204, 240, 275, 306
	Swine (Brazil)	2009–2011	24	0.331 (0.239, 0.445)	0	0
	Swine (Brazil)	2012–2020	67	0.217 (0.198, 0.239)	159	87, 159, 204, 240
	Swine Global (except Brazil)	2009–2019	130	0.256 (0.235, 0.278)	179	3, 86, 136, 179, 200, 240, 327
	Swine Global (except Brazil)	2009–2011	43	0.344 (0.283, 0.413)	0	240
	Swine Global (except Brazil)	2012–2019	87	0.240 (0.218, 0.262)	179	3, 86, 136, 158, 179, 327
NA	Human (Brazil)*	2009–2021	500	0.268 (0.248, 0.289)	129	13, 80, 82, 85, 86, 129, 229, 250, 321, 403, 412, 459
	Swine (Brazil)	2009–2020	71	0.220 (0.199, 0.242)	53	42, 53, 232
	Swine (Brazil)	2009–2011	16	0.258 (0.156, 0.396)	0	0
	Swine (Brazil)	2012–2020	55	0.217 (0.196, 0.239)	53	42, 53, 232
	Swine Global (except Brazil)	2009–2019	113	0.233 (0.210, 0.257)	0	3, 83, 232, 321, 367, 442, 469
	Swine Global (except Brazil)	2009–2011	24	0.425 (0.316, 0.555)	0	0
	Swine Global (except Brazil)	2012–2019	89	0.225 (0.202, 0.250)	0	3, 44, 83, 232, 321, 367, 442, 469

Since phylodynamic analyses indicated a different pattern of H1N1pdm09 evolution in swine in Brazil between 2009–2011 and 2012–2020, swine datasets were separated by collection date. We observed a decreasing trend of the global dN/dS rate ratio through time, suggesting higher ratios for the exponential phase of population growth (2009–2011) and smaller ratios during the stable phase (2012–2020) (Figures 4E,F). However, the small sample size in the 2009–2011 alignment resulted in a wider confidence interval. Genetic distance analyses suggested higher diversity of H1N1pdm09 swine strains sampled between 2012 and 2020. The temporal tendency on the global dN/dS (supported by the 95% confidence interval) is also evidenced by the analysis of H1N1pdm09 swine sequences isolated globally. These results demonstrated a clear difference in the evolution of H1N1pdm09 in swine over the two periods.

To determine whether different individual sites were under episodic or pervasive positive selection between 2009–2011 and 2012–2020, we analyzed all datasets using the mixed-effects model of evolution (MEME) algorithm. Our results showed a greater number of sites with statistically significant signals of positive selection for H1pdm09 and N1pdm09 in the period between 2012–2020. On the swine H1pdm09 alignments from Brazil, residues 87, 159, 204, and 240 were found to be under positive selection only for the 2012–2020 period, suggesting that these may be important sites associated with the host immune response (Table 1). The same pattern was observed for the N1pdm09 isolated among pigs in Brazil in which residues 45, 53 and 232 demonstrated a signal of positive selection during the 2012–2020 period which may associate with immune escape or immune driven selection. Most of these sites have never been detected in positive selection analysis in previous studies (Furuse et al., 2010; Joseph et al., 2017; Al Khatib et al., 2018).

## Discussion

This study expands our understanding about the extensive diversity of H1N1pdm09 circulating among swine populations in Brazil. Phylodynamic analyses revealed that HA and NA segments were repeatedly introduced into swine in Brazil since the beginning of the 2009 pandemic. In total, we identified 30 HA and NA separate human-to-swine spillovers. Despite the occurrence of numerous potential human-to-swine transmissions of H1N1pdm09, the majority of these introductions (87% for HA and 80% for NA) did not result in onward transmission. However, in a few cases, H1N1pdm09 continued to be successfully transmitted within swine populations in Brazil. Once established, these transmission chains persisted for several years, with an average duration of spillover persistence reaching approximately 7 years. These findings emphasize the frequency of human-swine contact and suggest that, despite the large number of transmissions, the swine immune system, acting as a species-barrier, might play a crucial role in maintaining the continuity of transmission chains (Nelson et al., 2012; Holzer et al., 2019; Martini et al., 2022).

Most of the human-to-swine H1N1pdm09 introductions in Brazil occurred before 2012. The evolutionary dynamics of the HA and NA segments during this period were shaped by several factors, including: (i) the occurrence of new introductions of H1N1pdm09 from humans into swine, (ii) pig-to-pig transmission of H1N1pdm09, and (iii) dead-end introductions. This timeframe coincided with an exponential increase in the relative genetic diversity of H1N1pdm09, as measured by the effective population size (Ne). This suggests that transmission from humans to a susceptible swine population, rather than swine-to-swine transmission, played a significant role in modulating the size of the viral population circulating among swine. We observed that most of the swine H1N1pdm09 sequences isolated in Brazil during this period resulted from single introductions (*p* < 0.001), indicating a high proportion of self-limited infections with apparent extinction in swine. This finding is likely associated with the increased incidence of H1N1pdm09 among humans, where a higher IAV burden in the human population may lead to more interspecies spillover events (Manito et al., 2017). Furthermore, viruses isolated during this phase exhibited higher global dN/dS rate ratios but did not show any positively selected sites. These results are likely a consequence of the rapid increase in relative genetic diversity in the absence of strong selective pressure, following the invasion of a naïve host population. Similar patterns were observed during the initial years of the human H1N1pdm09 pandemic (Su et al., 2015; Ma et al., 2020).

Following the exponential phase, the period from 2012 to 2020 exhibited a balance between H1N1pdm09 swine-to-swine transmissions and extinctions. Only a limited number of new human-to-swine introductions were identified during this period, and most of them did not lead to sustained transmission in swine. This decline in human-to-swine H1N1pdm09 transmissions may be attributed to the increasing vaccination coverage in human population in Brazil, resulting in reduced circulation of the virus and subsequently decreasing interspecies spillover events (Ministério da Saúde. Brasil. Secretaria de Vigilância em Saúde, 2009; World Health Organization, 2009). The decrease in dN/dS rate ratios observed during this phase may indicate the presence of selective constraints to maintain the functional fitness of the viruses circulating among swine. Additionally, the higher number of sequences isolated between 2012 and 2020 clustering within phylogenetic clades supports the notion that this phase is predominantly sustained by swine-to-swine transmissions.

Phylodynamic analyses revealed a small but pronounced decrease in the relative genetic diversity of H1N1pdm09, particularly in the HA gene, since 2019 (Figure 4). Despite the wide confidence intervals, this pattern may indicate an accumulated effect of the intensification of swine vaccination from 2017 onwards. In 2014, a monovalent H1N1pdm09 inactivated vaccine was licensed in Brazil, and, to date, it is the only commercially available vaccine for use in the country (van Reeth and Ma, 2012; Mancera Gracia et al., 2020; Fraiha et al., 2021). In addition, autogenous vaccines are also allowed for use but only on the target herd or adjacent properties (Chen et al., 2012) It is possible that the pattern we observe in the data is associated with a reduction in H1N1pdm09 circulation in pigs due to the efficacy of vaccine in swine. Alternatively, the decrease in relative genetic diversity could be explained by the replacement of the H1N1pdm09 by another swine IAV strain alongside the eradication of some of the diverse H1N1pdm09 clades through vaccination, however, more genomic surveillance data and antigenic characterization data are needed to understand how diversity and vaccination efforts impact IAV circulation.

Phylogenetic and phylodynamic analysis of IAV can benefit animal health guiding vaccination programs in swine through identifying circulating genetic groups and when those groups are changing in ways that may reduce vaccine efficacy. In addition, it provides an opportunity to track viral diversity, onward transmission, and spatial dissemination of viral genetic clades. This study elucidated the extent of the human H1N1pdm09 transmissions to Brazilian pigs through phylogenetic analysis. We demonstrated how human-to-swine transmission has changed the epidemiology of IAV in Brazil. Specifically, we demonstrated that at least 30 distinct genetic clades derived from different human influenza seasons have entered the swine population. Continued genomic assessment and antigenic characterization of these H1N1pdm09 viruses are required to determine how best to control transmission and determine how reassortment is affecting IAV population dynamics among swine in Brazil.

## Data availability statement

The datasets presented in this study can be found in online repositories. The names of the repository/repositories and accession number(s) can be found in the article/Supplementary material.

## Author contributions

DJ, CT, TA, and RS contributed to conception and design of the study. DG, VH, MC, CT, and RS performed the sample preparation and sequencing. DJ and CT organized the database and performed the analysis. DJ wrote the first draft of the manuscript. TA and AB wrote sections of the manuscript. All authors contributed to the article and approved the submitted version.

## Funding

This research was funded by EMBRAPA, grant number SEG 22.16.05.004.00.02 and USDA-ARS (contract 58-5030-0-055-F, ARS project number 5030-32000-231-000D). The APC was funded by ARS project number 58-5030-0-055-F. CT is a postdoc student (FUNARBE/ARS/USDA) number 13856. VH has a grant from Coordination for the Improvement of Higher Education Personnel (CAPES). The National Institute of Allergy and Infectious Diseases, National Institutes of Health, Department of Health and Human Services [contract number 75N93021C00015].

## Conflict of interest

The authors declare that the research was conducted in the absence of any commercial or financial relationships that could be construed as a potential conflict of interest.

## Publisher’s note

All claims expressed in this article are solely those of the authors and do not necessarily represent those of their affiliated organizations, or those of the publisher, the editors and the reviewers. Any product that may be evaluated in this article, or claim that may be made by its manufacturer, is not guaranteed or endorsed by the publisher.
