# Supplementary material for: Human-to-swine introductions and onward transmission of 2009 H1N1 pandemic influenza viruses in Brazil
Source: Front Microbiol. 2023 Aug 8;14:1243567. doi: 10.3389/fmicb.2023.1243567 (PMC10442540; doi:10.3389/fmicb.2023.1243567)
Supplement: Supplementary file 1 [file Table_1.docx]

Supplementary Material

Human-to-swine introductions and onward transmission of 2009 H1N1 pandemic influenza viruses in Brazil

**Dennis Maletich Junqueira1, Caroline Tochetto2, Tavis K. Anderson3, Danielle Gava², Vanessa Haach4, Maurício E. Cantão2, Amy L. Vincent Baker3, Rejane Schaefer²***

*** Correspondence:** Rejane Schaefer: rejane.schaefer@embrapa.br

**Supplementary Table 1.** Number and sequence composition of all the H1pdm09 and N1pdm09 clades and dead-end introductions identified in the maximum likelihood phylogenetic analyses.

| **Gene** | **Introduction** | **N** | **Branch Confidence (SH-aLRT/UFB)** | **Sequence** |
| --- | --- | --- | --- | --- |
| HA | **Clade 01** | 3 | 98.3/100 | A/Swine/Brazil/G3P1/KP027601/2013/H1N1 |
|  |  |  |  | A/Swine/Brazil/194153/MT041093/2015/H1N1 |
|  |  |  |  | A/Swine/Brazil/18/KM496987/2012/H1N1 |
|  | **Clade 02** | 5 | 99.2/100 | A/Swine/Brazil/281182/MT041109/2018/H1N1 |
|  |  |  |  | A/Swine/Brazil/52217/MT068751/2017/H1N1 |
|  |  |  |  | A/Swine/Brazil/11819/MW772635/2019/H1N1 |
|  |  |  |  | A/Swine/Brazil/17919/MW772917/2019/H1N1* |
|  |  |  |  | A/Swine/Brazil/12419/MW772516/2019/H1N1* |
|  | **Clade 03** | 3 | 99.2/98 | A/Swine/Brazil/1073A/KF683614/2010/H1N1 |
|  |  |  |  | A/Swine/Brazil/915/MH559923/2014/H1N1 |
|  |  |  |  | A/Swine/Brazil/21515/MH559915/2015/H1N1 |
|  | **Clade 04** | 42 | 99.6/100 | A/Swine/Brazil/52517/MT041141/2017/H1N1 |
|  |  |  |  | A/Human/Brazil/28600/EPI1838867/2020/H1N2 (Human) |
|  |  |  |  | A/Swine/Brazil/35717/MT041125/2017/H1N1 |
|  |  |  |  | A/Swine/Brazil/99143/MH560011/2014/H1N1 |
|  |  |  |  | A/Swine/Brazil/2515/MH559931/2015/H1N1 |
|  |  |  |  | A/Swine/Brazil/12019/MW772643/2019/H1N1 |
|  |  |  |  | A/Swine/Brazil/28151/MH559939/2015/H1N2 |
|  |  |  |  | A/Swine/Brazil/7220/MW772603/2020/H1N1 |
|  |  |  |  | A/Swine/Brazil/28152/MH559947/2015/H1N2 |
|  |  |  |  | A/Swine/Brazil/28154/MH559955/2015/H1N1 |
|  |  |  |  | A/Swine/Brazil/3315/MH559851/2015/H1N1 |
|  |  |  |  | A/Swine/Brazil/45152/MH559971/2015/H1N1 |
|  |  |  |  | A/Swine/Brazil/6115/MT041149/2015/H1N1* |
|  |  |  |  | A/Swine/Brazil/5215/MH559979/2015/H1N1* |
|  |  |  |  | A/Swine/Brazil/35152/MT041117/2015/H1N1* |
|  |  |  |  | A/Swine/Brazil/8419/MW772865/2019/H1N1 |
|  |  |  |  | A/Swine/Brazil/1220/MW772763/2020/H1N1 |
|  |  |  |  | A/Swine/Brazil/7020/MW772801/2020/H1N1 |
|  |  |  |  | A/Swine/Brazil/8319/MW772858/2019/H1N1 |
|  |  |  |  | A/Swine/Brazil/76192/MW772611/2019/H1N2* |
|  |  |  |  | A/Swine/Brazil/76191/MW772821/2019/H1N2* |
|  |  |  |  | A/Swine/Brazil/3616/MH559875/2016/H1N1 |
|  |  |  |  | A/Swine/Brazil/65152/MH560003/2015/H1N2 |
|  |  |  |  | A/Swine/Brazil/3161/MH559899/2015/H1N1 |
|  |  |  |  | A/Swine/Brazil/3516/MH559867/2016/H1N1 |
|  |  |  |  | A/Swine/Brazil/24618416/MT068805/2018/H1N1 |
|  |  |  |  | A/Swine/Brazil/5716/MT068783/2016/H1N1 |
|  |  |  |  | A/Swine/Brazil/134161/MT068648/2016/H1N1 |
|  |  |  |  | A/Swine/Brazil/419/MW772731/2019/H1N2 |
|  |  |  |  | A/Swine/Brazil/104184/MT041077/2018/H1N1 |
|  |  |  |  | A/Swine/Brazil/66192/MW772563/2018/H1N1 |
|  |  |  |  | A/Swine/Brazil/6920/MW772587/2020/H1N1 |
|  |  |  |  | A/Swine/Brazil/13519/MW772667/2019/H1N1 |
|  |  |  |  | A/Swine/Brazil/299172/MT068812/2017/H1N2 |
|  |  |  |  | A/Swine/Brazil/520/MW772737/2019/H1N2 |
|  |  |  |  | A/Swine/Brazil/620/MW772743/2019/H1N2 |
|  |  |  |  | A/Swine/Brazil/299171/MT068715/2017/H1N2 |
|  |  |  |  | A/Swine/Brazil/279182/MT041061/2018/H1N1** |
|  |  |  |  | A/Swine/Brazil/279181/MT041053/2018/H1N1** |
|  |  |  |  | A/Swine/Brazil/70193/MW772476/2018/H1N2* |
|  |  |  |  | A/Swine/Brazil/70192/MW772794/2018/H1N2* |
|  |  |  |  | A/Swine/Brazil/70191/MW772789/2018/H1N2* |
|  | **Dead-End 01** | 1 | - | A/Swine/Brazil/JQ666851/2009/H1N1 |
|  | **Dead-End 02** | 3 | - | A/Swine/Brazil/2/JQ666846/2009/H1N1* |
|  | **Dead-End 02** |  |  | A/Swine/Brazil/JQ666845/2009/H1N1* |
|  | **Dead-End 02** |  |  | A/Swine/Brazil/JQ666847/2009/H1N1* |
|  | **Dead-End 03** | 1 | - | A/Swine/Brazil/18/JQ666862/2010/H1N1 |
|  | **Dead-End 04** | 1 | - | A/Swine/Brazil/60141/MH559891/2014/H1N1 |
|  | **Dead-End 05** | 2 | - | A/Swine/Brazil/13219/MW772885/2019/H1N1 |
|  | **Dead-End 05** |  |  | A/Swine/Brazil/215/MH559835/2014/H1N1 |
|  | **Dead-End 06** | 1 | - | A/Swine/Brazil/66/KM496995/2011/H1N1 |
|  | **Dead-End 07** | 1 | - | A/Swine/Brazil/9018/MT041173/2018/H1N1 |
|  | **Dead-End 08** | 1 | - | A/Swine/Brazil/11/JQ666855/2009/H1N1 |
|  | **Dead-End 09** | 2 | - | A/Swine/Brazil/263/KM497003/2012/H1N1 |
|  | **Dead-End 09** |  |  | A/Swine/Brazil/61141/MH559987/2014/H1N1 |
|  | **Dead-End 10** | 1 | - | A/Swine/Brazil/14/JQ666858/2009/H1N1 |
|  | **Dead-End 11** | 2 | - | A/Swine/Brazil/19/JQ666863/2010/H1N1* |
|  | **Dead-End 11** |  |  | A/Swine/Brazil/20/JQ666864/2010/H1N1* |
|  | **Dead-End 12** | 2 | - | A/Swine/Brazil/12/JQ666856/2009/H1N1 |
|  | **Dead-End 12** |  |  | A/Swine/Brazil/13/JQ666857/2009/H1N1 |
|  | **Dead-End 13** | 2 | - | A/Swine/Brazil/JQ666849/2009/H1N1* |
|  | **Dead-End 13** |  |  | A/Swine/Brazil/6/JQ666850/2009/H1N1* |
|  | **Dead-End 14** | 1 | - | A/Swine/Brazil/15/JQ666859/2009/H1N1 |
|  | **Dead-End 15** | 2 | - | A/Swine/Brazil/16/JQ666860/2009/H1N1* |
|  | **Dead-End 15** |  |  | A/Swine/Brazil/17/JQ666861/2009/H1N1* |
|  | **Dead-End 16** | 1 | - | A/Swine/Brazil/12A/JF421756/2010/H1N1 |
|  | **Dead-End 17** | 1 | - | A/Swine/Brazil/132/KM497008/2009/H1N1 |
|  | **Dead-End 18** | 1 | - | A/Swine/Brazil/4/JQ666848/2009/H1N1 |
|  | **Dead-End 19** | 1 | - | A/Swine/Brazil/35617/MT068819/2017/H1N2 |
|  | **Dead-End 20** | 3 | - | A/Swine/Brazil/8/JQ666852/2009/H1N1 |
|  | **Dead-End 20** |  |  | A/Swine/Brazil/9/JQ666853/2009/H1N1 |
|  | **Dead-End 20** |  |  | A/Swine/Brazil/10/JQ666854/2009/H1N1 |
|  | **Dead-End 21** | 1 | - | A/Swine/Brazil/13319/MW772659/2019/H1N1 |
|  | **Dead-End 22** | 1 | - | A/Swine/Brazil/13119/MW772651/2019/H1N1 |
|  | **Dead-End 23** | 2 | - | A/Swine/Brazil/911848/MT041181/2018/H1N1 |
|  | **Dead-End 23** |  |  | A/Swine/Brazil/52817/MT068776/2017/H1N1 |
|  | **Dead-End 24** | 2 | - | A/Swine/Brazil/62183/MT041157/2018/H1N1 |
|  | **Dead-End 24** |  |  | A/Swine/Brazil/62184/MT041165/2018/H1N1 |
|  | **Dead-End 25** | 1 | - | A/Swine/Brazil/56194/MW772539/2018/H1N1 |
|  | **Dead-End 26** | 2 | - | A/Swine/Brazil/63194/MW772547/2019/H1N1 |
|  | **Dead-End 26** |  |  | A/Swine/Brazil/63197/MW772555/2019/H1N1 |
| NA | **Clade 01** | 5 | 99/92 | A/Swine/Brazil/1073A/KF683616/2010/H1N1 |
|  |  |  |  | A/Swine/Brazil/24618416/NA/2018/H1N1 |
|  |  |  |  | A/Swine/Brazil/13219/MW772887/2019/H1N1 |
|  |  |  |  | A/Swine/Brazil/279182/NA/2018/H1N1* |
|  |  |  |  | A/Swine/Brazil/279181/NA/2018/H1N1* |
|  | **Clade 02** | 5 | 99.2/100 | A/Swine/Brazil/8419/MW772867/2019/H1N1 |
|  |  |  |  | A/Swine/Brazil/1220/MW772765/2020/H1N1 |
|  |  |  |  | A/Swine/Brazil/7020/MW772803/2020/H1N1 |
|  |  |  |  | A/Swine/Brazil/8319/MW772860/2019/H1N1 |
|  |  |  |  | A/Human/Brazil/10835/EPI1853137/2021/H1N1 (Human) |
|  | **Clade 03** | 13 | 95.2/100 | A/Swine/Brazil/13519/MW772669/2019/H1N1 |
|  |  |  |  | A/Swine/Brazil/3616/MH559877/2016/H1N1 |
|  |  |  |  | A/Swine/Brazil/99143/MH560013/2014/H1N1 |
|  |  |  |  | A/Swine/Brazil/12019/MW772645/2019/H1N1 |
|  |  |  |  | A/Swine/Brazil/28154/MH559957/2015/H1N1 |
|  |  |  |  | A/Swine/Brazil/2515/MH559933/2015/H1N1 |
|  |  |  |  | A/Swine/Brazil/7220/MW772605/2020/H1N1 |
|  |  |  |  | A/Swine/Brazil/3315/MH559853/2015/H1N1 |
|  |  |  |  | A/Swine/Brazil/35152/NA/2015/H1N1 |
|  |  |  |  | A/Swine/Brazil/45152/MH559973/2015/H1N1 |
|  |  |  |  | A/Swine/Brazil/6115/NA/2015/H1N1* |
|  |  |  |  | A/Swine/Brazil/5215/EPI1829588/2015/H1N1* |
|  |  |  |  | A/Swine/Brazil/5215/MH559981/2015/H1N1* |
|  | **Clade 04** | 6 | 95.8/99 | A/Swine/Brazil/11819/MW772637/2019/H1N1 |
|  |  |  |  | A/Swine/Brazil/17919/MW772919/2019/H1N1* |
|  |  |  |  | A/Swine/Brazil/12419/MW772518/2019/H1N1* |
|  |  |  |  | A/Swine/Brazil/13619/MW772677/2019/H1N1 |
|  |  |  |  | A/Swine/Brazil/52217/NA/2017/H1N1* |
|  |  |  |  | A/Swine/Brazil/281182/NA/2018/H1N1* |
|  | **Clade 05** | 3 | 100/100 | A/Swine/Brazil/G3P1/KP027603/2013/H1N1 |
|  |  |  |  | A/Swine/Brazil/194153/NA/2015/H1N1 |
|  |  |  |  | A/Swine/Brazil/18/KM496989/2012/H1N1 |
|  | **Clade 06** | 5 | 100/100 | A/Swine/Brazil/61141/MH559989/2014/H1N1 |
|  |  |  |  | A/Swine/Brazil/263/KM497005/2012/H1N1 |
|  |  |  |  | A/Swine/Brazil/6920/MW772589/2020/H1N1 |
|  |  |  |  | A/Swine/Brazil/66192/MW772565/2018/H1N1 |
|  |  |  |  | A/Swine/Brazil/104184/NA/2018/H1N1 |
|  | **Dead-End 01** | 1 | - | A/Swine/Brazil/215/MH559837/2014/H1N1 |
|  | **Dead-End 02** | 1 | - | A/Swine/Brazil/66/KM496997/2011/H1N1 |
|  | **Dead-End 03** | 2 | - | A/Swine/Brazil/3161/MH559901/2015/H1N1 |
|  | **Dead-End 03** |  |  | A/Swine/Brazil/3516/MH559869/2016/H1N1 |
|  | **Dead-End 04** | 1 | - | A/Swine/Brazil/60141/MH559893/2014/H1N1 |
|  | **Dead-End 05** | 1 | - | A/Swine/Brazil/9018/NA/2018/H1N1 |
|  | **Dead-End 06** | 2 | - | A/Swine/Brazil/5716/NA/2016/H1N1 |
|  | **Dead-End 06** |  |  | A/Swine/Brazil/21515/MH559917/2015/H1N1 |
|  | **Dead-End 07** | 1 | - | A/Swine/Brazil/52517/NA/2017/H1N1 |
|  | **Dead-End 08** | 1 | - | A/Swine/Brazil/74192/MW772816/2019/H1N1 |
|  | **Dead-End 09** | 1 | - | A/Swine/Brazil/14/JQ666879/2009/H1N1 |
|  | **Dead-End 10** | 1 | - | A/Swine/Brazil/4/JQ666869/2009/H1N1 |
|  | **Dead-End 11** | 1 | - | A/Swine/Brazil/12A/KM507542/2010/H1N1 |
|  | **Dead-End 12** | 2 | - | A/Swine/Brazil/35717/NA/2017/H1N1 |
|  | **Dead-End 12** |  |  | A/Swine/Brazil/915/MH559925/2014/H1N1 |
|  | **Dead-End 13** | 2 | - | A/Swine/Brazil/JQ666867/2009/H1N1* |
|  | **Dead-End 13** |  |  | A/Swine/Brazil/JQ666866/2009/H1N1* |
|  | **Dead-End 14** | 2 | - | A/Swine/Brazil/16/JQ666881/2009/H1N1 |
|  | **Dead-End 14** |  |  | A/Swine/Brazil/17/JQ666882/2009/H1N1 |
|  | **Dead-End 15** | 2 | - | A/Swine/Brazil/20/JQ666885/2010/H1N1* |
|  | **Dead-End 15** |  |  | A/Swine/Brazil/19/JQ666884/2010/H1N1* |
|  | **Dead-End 16** | 3 | - | A/Swine/Brazil/8/JQ666873/2009/H1N1* |
|  | **Dead-End 16** |  |  | A/Swine/Brazil/9/JQ666874/2009/H1N1* |
|  | **Dead-End 16** |  |  | A/Swine/Brazil/10/JQ666875/2009/H1N1* |
|  | **Dead-End 17** | 1 | - | A/Swine/Brazil/15/JQ666880/2009/H1N1 |
|  | **Dead-End 18** | 1 | - | A/Swine/Brazil/18/JQ666883/2010/H1N1 |
|  | **Dead-End 19** | 2 | - | A/Swine/Brazil/62184/NA/2018/H1N1 |
|  | **Dead-End 19** |  |  | A/Swine/Brazil/62183/NA/2018/H1N1 |
|  | **Dead-End 20** | 2 | - | A/Swine/Brazil/52817/NA/2017/H1N1 |
|  | **Dead-End 20** |  |  | A/Swine/Brazil/911848/NA/2018/H1N1 |
|  | **Dead-End 21** | 1 | - | A/Swine/Brazil/13119/MW772653/2019/H1N1 |
|  | **Dead-End 22** | 1 | - | A/Swine/Brazil/13319/MW772661/2019/H1N1 |
|  | **Dead-End 23** | 2 | - | A/Swine/Brazil/56194/MW772541/2018/H1N1* |
|  | **Dead-End 23** |  |  | A/Human/Brazil/8539/EPI1312652/2018/H1N1*(Human) |
|  | **Dead-End 24** | 2 | - | A/Swine/Brazil/63194/MW772549/2019/H1N1* |
|  | **Dead-End 24** |  |  | A/Swine/Brazil/63197/MW772557/2019/H1N1* |

* duplicate sequences

UFB, ultra fast boostrap
