# Supplementary material for: Human-to-swine introductions and onward transmission of 2009 H1N1 pandemic influenza viruses in Brazil
Source: Front Microbiol. 2023 Aug 8;14:1243567. doi: 10.3389/fmicb.2023.1243567 (PMC10442540; doi:10.3389/fmicb.2023.1243567)
Supplement: Supplementary file 3 [file Image_1.pdf]

Supplementary Material

Human-to-swine introductions and onward transmission of 2009 H1N1 pandemic influenza viruses in Brazil

Dennis Maletich Junqueira1, Caroline Tochetto2, Tavis K. Anderson3, Danielle Gava², Vanessa Haach4, Maurício E. Cantão2, Amy L. Vincent Baker3, Rejane Schaefer²*

*** Correspondence:** Rejane Schaefer: rejane.schaefer@embrapa.br

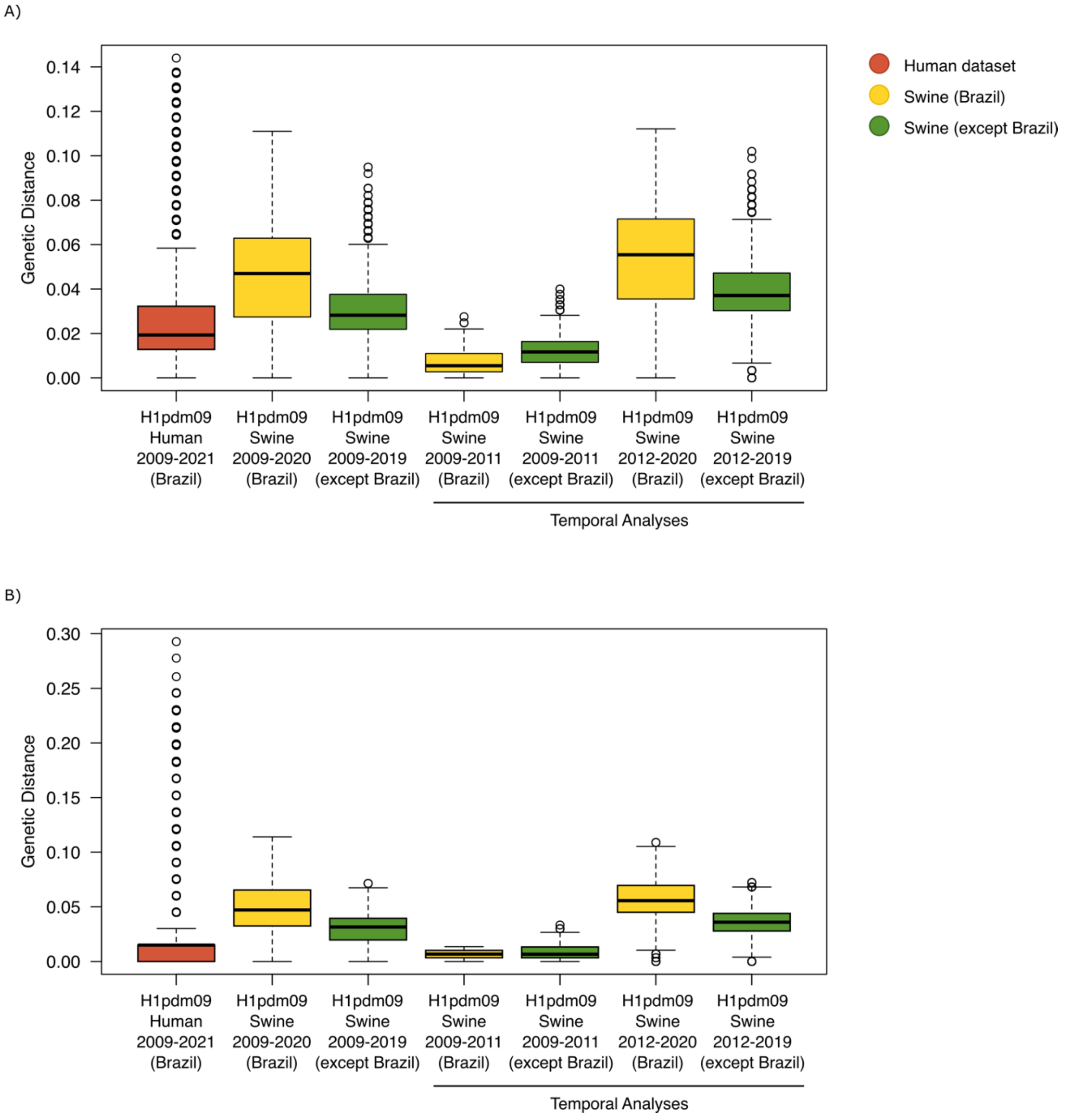

Supplementary Figure 1. Box plots showing the genetic distances within and between seven clusters of H1pdm9 HA (A) and N1pdm01 NA (B). In each box plot, the box shows the interquartile range (IQR) of the data. The IQR is defined as the difference between the 75th percentile and the 25th percentile. The whiskers represent variability outside the upper and lower quartiles. The solid line through the box represents the median pairwise genetic distance.
